# Three-dimensional nanoscale molecular imaging by extreme ultraviolet laser ablation mass spectrometry

**DOI:** 10.1038/ncomms7944

**Published:** 2015-04-23

**Authors:** Ilya Kuznetsov, Jorge Filevich, Feng Dong, Mark Woolston, Weilun Chao, Erik H. Anderson, Elliot R. Bernstein, Dean C. Crick, Jorge J. Rocca, Carmen S. Menoni

**Affiliations:** 1 NSF Center for Extreme Ultraviolet Science and Technology, Colorado State University, Fort Collins, Colorado 80523, USA; 2 Department of Electrical and Computer Engineering, Colorado State University, Fort Collins, Colorado 80523, USA; 3 Department of Chemistry, Colorado State University, Fort Collins, Colorado 80523, USA; 4 Center for X-Ray Optics, Lawrence Berkeley Laboratory, Berkeley, CA 94720, USA; 5 Department of Microbiology, Immunology and Pathology, Colorado State University, Fort Collins, Colorado 80523, USA; 6 Department of Physics, Colorado State University, Fort Collins, Colorado 80523, USA

## Abstract

Analytical probes capable of mapping molecular composition at the nanoscale are of critical importance to materials research, biology and medicine. Mass spectral imaging makes it possible to visualize the spatial organization of multiple molecular components at a sample's surface. However, it is challenging for mass spectral imaging to map molecular composition in three dimensions (3D) with submicron resolution. Here we describe a mass spectral imaging method that exploits the high 3D localization of absorbed extreme ultraviolet laser light and its fundamentally distinct interaction with matter to determine molecular composition from a volume as small as 50 zl in a single laser shot. Molecular imaging with a lateral resolution of 75 nm and a depth resolution of 20 nm is demonstrated. These results open opportunities to visualize chemical composition and chemical changes in 3D at the nanoscale.

## Abstract (web summary)

Mass spectral analysis is used to map the composition of materials and surfaces in numerous fields. Here, the authors report a mass spectral technique based on extreme ultraviolet laser ablation that allows three-dimensional imaging of chemical composition in addition to giving highly sensitive nanoscale resolution.

## Graphical abstract

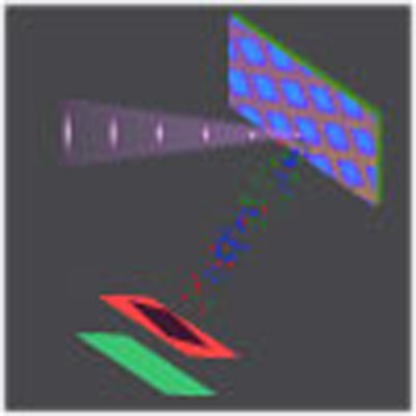

Mass spectral imaging (MSI) is an essential tool for surface analysis. MSI has achieved high sensitivity and spatial resolution in the identification and profiling of element-specific signatures from dopants and impurities in semiconductors, metals and dielectrics. The elemental specificity of MSI has also motivated studies in biology, medicine and environmental sciences in which fundamental molecular processes have been identified from characteristic signatures from metallic or isotopic tags^1^,^2^,^3^. Adapting MSI to molecular imaging has required the implementation of ionization strategies, which control molecular fragmentation^4^. In biochemistry and medicine, molecular MSI is used, for example, in studies of metabolic exchange in microorganisms^5^ and localization of drugs and metabolites in tissue^6^.

MSI typically uses a laser or an ion beam to ablate or sputter the sample's surface and desorb and ionize atoms and molecules. Spatially resolved mass detection of the ejected material allows one to construct two-dimensional (2D) maps of the spatial organization of multiple molecular components at the sample's surface. The leading molecular MSI methods are laser desorption ionization (LDI) and secondary ion mass spectrometry (SIMS)^4^. LDI MSI has been demonstrated using laser wavelengths ranging from the near infrared to the ultraviolet and pulse durations from nanoseconds to femtoseconds. The ablation, desorption and ionization mechanisms depend significantly on the laser pulse duration, fluence and the absorption characteristics of the material at the laser wavelength. For applications to organics, LDI MSI is most commonly implemented using nanosecond ultraviolet pulses. The relatively low absorption of ultraviolet light by organic samples requires the use of a highly absorbing matrix to promote absorption, desorption and ionization^7^,^8^. In ultraviolet LDI MSI, the diffraction limit of the ultraviolet illumination coupled with the properties of the matrix affect the lateral spatial resolution, which is typically ∼10 μm and at best ∼1 μm. In addition, it is difficult to obtain high depth resolution^9^. Matrix assisted ultraviolet LDI time-of-flight (TOF) MSI is used in biomedical imaging for its ability to detect large molecules (*m*/*z*>1,000) as lipids and proteins in tissue^9^,^10^,^11^,^12^.

SIMS uses energetic ion bombardment to erode the sample's surface. In this process, secondary ions are created from the top few monolayers through collision cascade events that extend tens of nanometres into the bulk depending on the material, the energy and type of the primary ions. For molecular imaging of organic solids, secondary ion TOF mass spectrometry (SIMS TOF) uses primary ions with energies of ∼10–30 keV and the dose at the sample is limited to <10^12^ cm^−2^ to minimize damage. SIMS TOF MSI is capable to image the 2D distribution of intact analyte molecules within a mass range of *m*/*z* ∼1,000 with typically ∼1-μm lateral resolution and independently depth profile molecular content with ∼10-nm depth resolution^13^,^14^. A higher lateral resolution of ∼60 nm has been demonstrated with SIMS TOF using 80 keV primary ions, although at the expense of molecular fragmentation and thus reduced mass range (*m*/*z* 184)^15^.

There are significant challenges for MSI to reach nanoscale 3D spatial resolution in the chemical imaging of organic samples. In matrix assisted ultraviolet LDI-TOF, the matrix/analyte interaction combined with fundamental wavelength limitations precludes the increase of lateral spatial resolution below ∼1 μm and at the same time makes it difficult to implement depth profiling. In SIMS TOF analyte, ion yield and molecular fragmentation limit the ability to achieve nanoscale lateral and depth resolution simultaneously.

In this article, we describe a new laser ablation and ionization MSI method that exploits the superior focusability of extreme ultraviolet (EUV) laser light, its shallow absorption depth and its distinct interactions with materials to map chemical composition of organic samples in 3D at the nanoscale. In this first demonstration of EUV laser ablation TOF MSI (EUV TOF), the method is shown to detect singly ionized intact analyte ions with a superior sensitivity of 0.01 amol within the mass range of up to *m*/*z* 400. Molecular composition across a sharp boundary is assessed with lateral resolution of 75 nm and a depth resolution of 20 nm. We exploited the high localization of the focused EUV light for 3D molecular imaging of a single *Mycobacterium smegmatis*.

## Results

### Concept and implementation of 3D chemical imaging by EUV laser ablation

**Figure 1:**
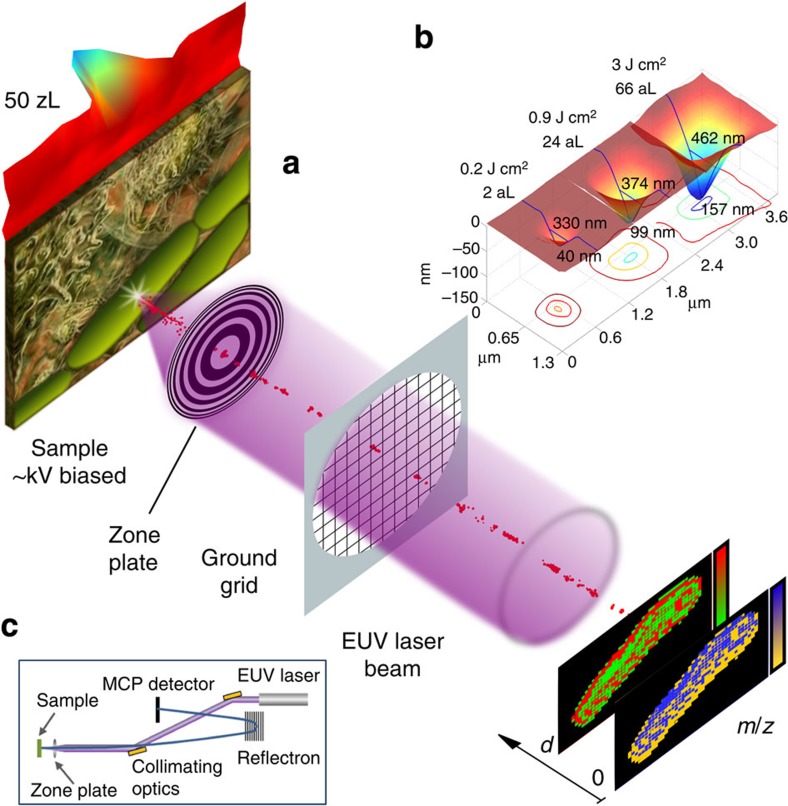
Extreme ultraviolet laser ablation mass spectrometry imaging concept. (**a**) Schematics showing the focused EUV laser beam ablating a sample to produce an ion stream that is analyzed by a TOF mass spectrometer. (**b**) Atomic force microscope (AFM) images of craters ablated in polymethyl methacrylate (PMMA) by a single EUV laser shot at different irradiation fluences. The smallest ablation crater has a volume of ∼50 zl. The craters show smooth profiles with no signs of thermal damage. (**c**) Schematic of the instrument set up including the collimating EUV laser optics, focusing zone plate and TOF spectrometer.

The concept of EUV TOF MSI is illustrated in Fig. 1a. Bright laser pulses from a compact 46.9-nm wavelength (*λ*) laser^16^ are focused into nanometre-sized spots to ablate craters a few nanometres deep on selected regions of the sample. The ions in the laser-created plasma are extracted and identified by their mass-to-charge ratio (*m*/*z*) using a TOF mass spectrometer. 3D composition images are constructed from the analysis of spatially resolved mass spectra obtained as the sample is displaced with respect to the focused laser beam.

The EUV MSI instrument is schematically shown in Fig. 1c. The EUV laser generates pulses of ∼10 μJ energy, 1.5 ns duration at a wavelength of *λ*=46.9 nm (ref 16). The laser output is collimated using a pair of grazing incidence toroidal mirrors to fully illuminate a zone plate (ZP) lens that focuses the laser beam onto the sample. The free standing ZP consists of concentric zones with outermost zone width of 200 nm and a central zone opening of 50-μm diameter that allows for ion extraction^17^. Positive ions from the EUV laser-produced nanoplasma are accelerated across a typically 6 kV potential difference and are injected into a TOF tube operated in reflectron mode. The mass resolution of the TOF detector was determined to be *m*/Δ*m*=1,100. For operation in imaging mode, the sample was mounted on x–y–z nanopositioners.

Atomic force microscope (AFM) images of craters ablated in polymethyl methacrylate (PMMA) with single EUV laser shots at different irradiation fluences are displayed in Fig. 1b. The profiles were obtained by placing the sample at the first-order focus of the ZP and attenuating the laser beam intensity by photoionization of argon in a variable pressure gas cell. The laser fluence necessary to ablate each of the craters is indicated in Fig. 1b. The ablated volume was varied from 66 to 2 al when decreasing the laser fluence by ∼15 × . Smaller craters can be created operating in the third order of the ZP (see Methods). The smallest crater ablated from which distinct analyte signatures were obtained has a volume of ∼50 zl (Fig. 1a; Supplementary Fig. S.2a). The AFM images of the ablated craters also show their profile is very smooth with no signs of thermal damage. This results from the prevalence of chain scission in the polymer by the EUV photons^18^.

### Demonstration of 3D molecular imaging at the nanoscale

A set of experiments was designed to benchmark the ability of EUV TOF MSI to detect intact molecular ions from relevant organic molecules, to determine its lateral and depth spatial resolution when imaging heterogenous samples, and to image molecular composition in 3D.

**Figure 2:**
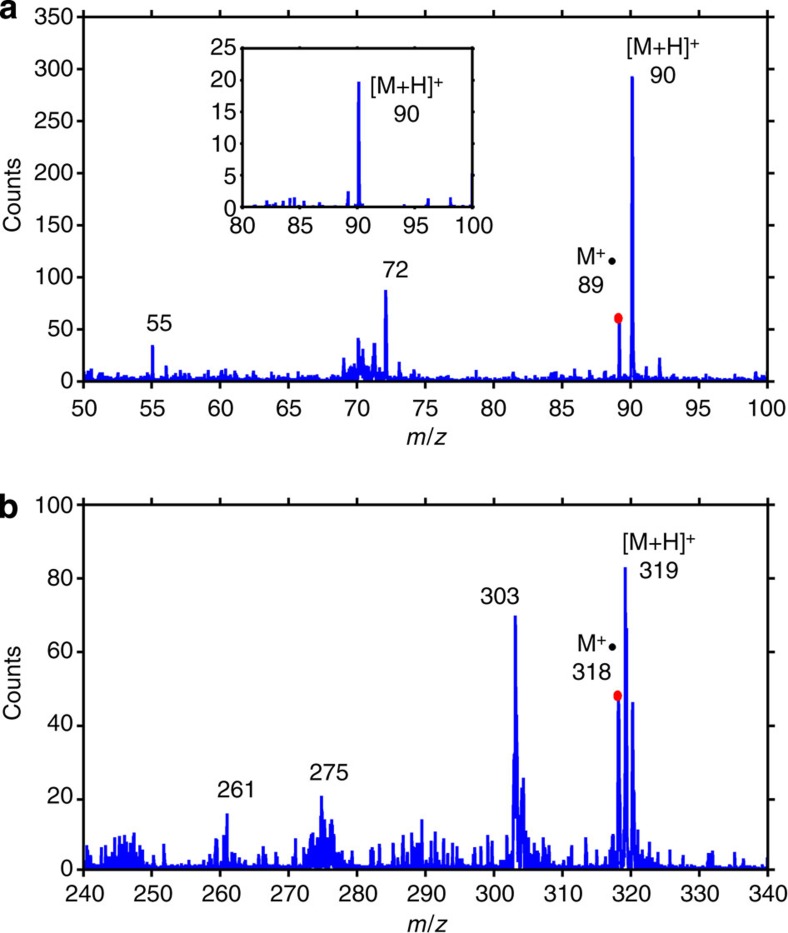
Single-shot mass spectra of organic analytes. (**a**) Mass spectrum of alanine obtained from an ablated analyte volume of 12.8 al in which the protonated molecule peak [M+H]^+^ and the radical M·^+^ are identified. The inset shows the amplitude of [M+H]^+^ is significantly above the noise floor when the ablated analyte volume is 50 zl. The alanine peak at *m*/*z* 72 corresponds to the loss of water [M+H-H_2_O]^+^ and the peak at *m*/*z* 55 to [M+H-CH_3_-NH_2_]^+^. (**b**) Mass spectrum of Nile red obtained from the same ablated analyte volume showing [M+H]^+^ and M·^+^ with *m*/*z* of 319 and 318, respectively.

The mass detection range and sensitivity were assessed from mass spectrometry experiments that used thin layer samples of the amino acid alanine (CH_3_CH(NH_2_)COOH, molecular mass: 89.09 Da, monoisotopic mass: 89.05 Da) and the organic dye Nile red (C_20_H_18_N_2_O_2_, molecular mass: 318.369 Da, monoisotopic mass: 318.14 Da). Single-shot mass spectra were obtained ablating the analyte placed at either the first- or the third-order focus of the ZP lens. The mass spectrum of alanine in Fig. 2a contains intense peaks that are identified as the protonated molecular ion [M+H]^+^ with *m*/*z* 90 and the radical M·^+^ with *m*/*z* 89. These signatures are well resolved even when ablating a 50-zl crater (Supplementary Fig. 2a). By taking the ratio of the ablated analyte mass in moles to the total counts within the area of [M+H]^+^, the sensitivity of EUV TOF is calculated to be 0.01 amol. The level of fragmentation was also calculated from the alanine spectrum as the ratio of the integrated counts in [M+H]^+^ to the total number of counts in the spectrum within 50≤*m*/*z*≤89 and found to be 1.1. The single-shot mass spectrum of Nile red is shown in Fig. 2b. This spectrum contains intense peaks at *m*/*z* 319 and 318, which correspond to [M+H]^+^ and M·^+^ and salient molecular fragments at *m*/*z* 303, 275 and 261. A comparison of the sensitivity and level of fragmentation of EUV TOF with SIMS TOF is shown in Supplementary Fig. 1 and summarized in Supplementary Table I. Details of this analysis, presented in the Supplementary Discussion show EUV TOF to be highly more sensitive.

**Figure 3:**
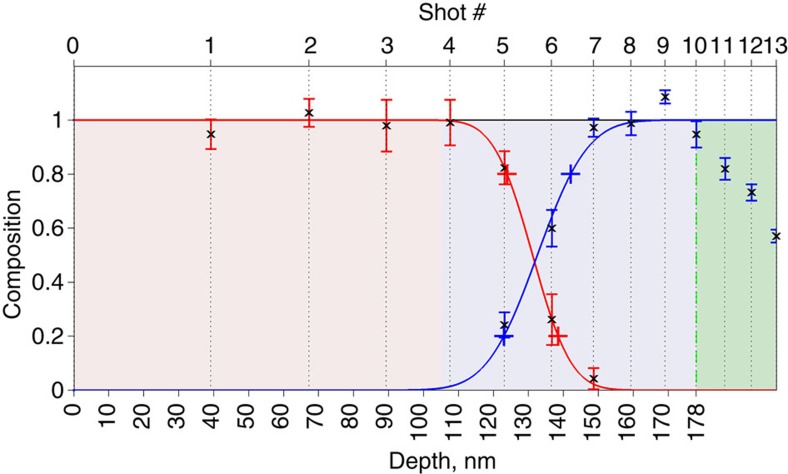
Variation of molecular composition versus depth. The average amplitude of the Nile red—in red—and alanine—in blue—peaks in the spectra of Fig. 2 is plotted versus ablation event number and ablation depth. After the 10th shot, the depth of the ablation craters remains practically unchanged indicating the ITO layer was reached. The depth scale was determined from atomic force measurements of the ablated craters. The error bars represent the variance in the amplitude for each analyte at each depth obtained from the 16 consecutive ablation events as described in Methods. The plus signs in the intensity profiles indicate the 20 and 80% levels from which the depth resolution is obtained.

Alanine and Nile red were selected to assess the depth resolution of EUV TOF MSI as their molecular signatures are in distinct *m*/*z* ranges. A sample consisting of a 105-nm thick layer of Nile red and a 73-nm thick layer of alanine deposited by evaporation onto an ITO-covered glass slide was used. A laser fluence of 0.13 J cm^−2^ was selected to ablate the organic layers but not the ITO-coated glass substrate. The depth profile of the organic bilayer structure was obtained by recording the mass spectrum from 13 consecutive laser shots fired on the same spot. For statistical purposes, the process was repeated on other sites arranged in a 4 × 4 array of points separated by 2.5 μm. Laser shot number was correlated to depth through independent AFM measurements of the ablated analyte profiles. Beyond the 10th ablation event the depth of the craters remained unchanged, indicating that the ITO layer was reached. The normalized abundance or composition profile (see Methods) of each of the analytes versus depth is plotted in Fig. 3. A depth resolution of 20 nm was determined from a fit of these profiles with a sigmoid function. This is the distance corresponding to the 20–80% change in the amplitude of the analyte signal across the interface.

**Figure 4:**
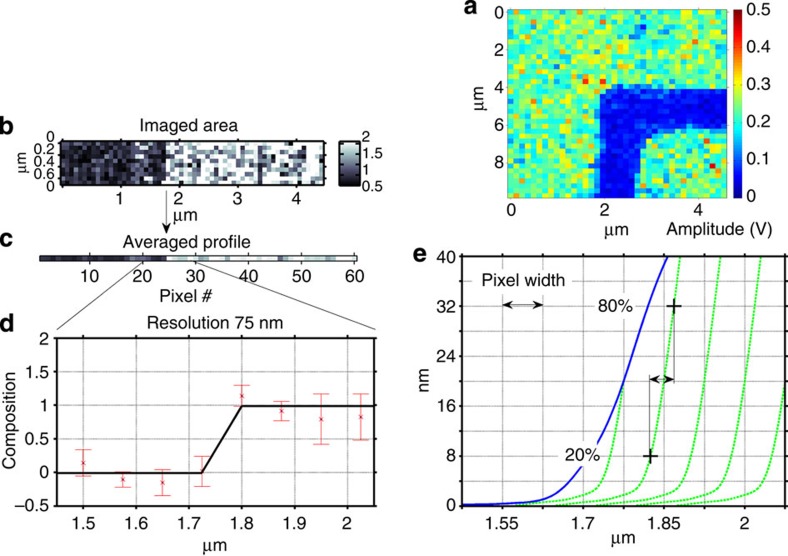
Demonstration of 2D nanoscale molecular imaging. (**a**) 2D ion image depicting the distribution of resist over a 10 × 10 μm^2^ region of the sample in which a trench was defined by photolithography. The pixel size in the image is 250 nm. The colour scale represents the resist content. (**b**) High resolution 2D ion image of the trench region showing in a grey scale the resist content. The pixel size is 75 nm. (**c**) Vertically averaged intensity profile over the region of the sample extending 4.5 μm. (**d**) The intensity lineout of this profile shows the 20–80% change occurs within one pixel. (**e**) Resist edge profiles obtained from AFM images before ablation (blue trace) and after consecutive ablation events (green trace). The + signs indicate the 20–80% rise in the ablation profiles is sub-pixel.

Nanoscale 2D MSI was demonstrated using a sample consisting of a ∼120-nm layer of resist containing a trench that exposed the ITO-coated glass substrate. In these experiments, a laser fluence of ∼0.43 J cm^−2^ was selected to ablate craters in the resist with a mean full width at half maximum (FWHM) diameter of 400 nm and a depth of 40 nm in a single-shot. A broad area image of the trench with pixel size of 250 nm is shown in Fig. 4a. Each pixel in the image plots the resist content, which was determined by averaging the amplitude of four characteristic resist fragments in the *m*/*z* range of 74–121. A region extending 4.5 μm across and 0.8 μm along the trench was imaged with higher resolution. In this experiment, consecutive mass spectra were obtained as the sample was displaced in 75 nm steps with respect to the focused laser spot. The 2D ion image of this region is shown in Fig. 4b. The grey scale indicates the resist content in each pixel calculated as in the broad area image of Fig. 4a. To assess the lateral resolution, the amplitude of each 10 pixel high column in the 2D ion image of Fig. 4b was averaged to create the average profile of Fig. 4c. Analysis of this profile shows the 20–80% change in amplitude occurs within one pixel, that is, 75 nm (Fig. 4d). AFM profiles of the resist edge before ablation (blue trace) and after consecutive ablation (green traces) are shown in Fig. 4e. The sharp edge of the ablation profile with sub-pixel 20–80% rise enables the demonstrated high spatial resolution in the imaging of organic materials.

**Figure 5:**
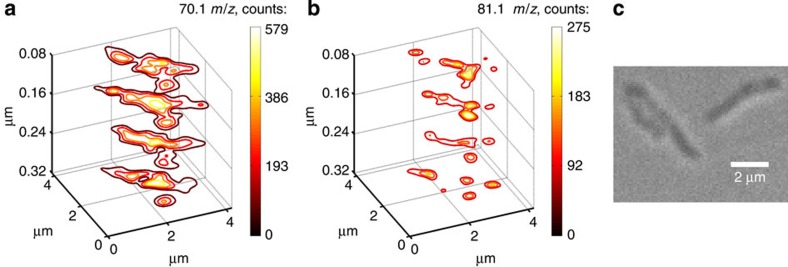
Demonstration of 3D molecular imaging. 3D ion image of a *Mycobacterium smegmatis*. The iso-lines show the distribution of two significant fragments, *m*/*z* 70.1 (**a**) and 81.1 (**b**) detected by single-shot EUV TOF. The image is constructed from spline-interpolated 0.3 × 0.3 × 0.08 μm^3^ voxels. A confocal microscope image of the sample is shown (**c**).

To demonstrate 3D molecular imaging, we used a sample consisting of *Mycobacterium smegmatis* bacteria deposited on an ITO-coated glass substrate. The laser fluence ∼0.5 J cm^−2^ was selected to ablate only the microorganisms and not the ITO-coated substrate. Mass spectra were obtained from a voxel size 300 nm in diameter and 80 nm in depth. Figure 5 plots the distribution of two dominant ions in the spectra with *m*/*z* 70.1 and 81.1. The traces are iso-lines corresponding to different peak intensities. The evolution of a bacterium's profile is clearly seen in Fig. 5a. The mass resolution of the TOF is not sufficient to univocally identify these ions.

## Discussion

The high lateral, 75 nm, and depth, 20 nm, resolution and high sensitivity of EUV TOF demonstrated in this work are the result of the unique properties of EUV light and of its interaction with organic solids. The use of *λ*=46.9-nm laser light for MSI is optimal because of the following: (i) it can be focused into spots of ∼100 nm, significantly smaller than those obtained with ultraviolet light resulting in superior lateral resolution^19^; (ii) its absorption depth is extremely shallow, that is, ∼20 nm^20^, thus, making it possible to ablate craters a few nanometres deep by controlling the laser fluence at the sample (Fig. 1a; Supplementary Fig. 2a); and (iii) the EUV photons can directly photoionize organic molecules. Moreover, the inherent high absorption of organic materials at *λ*=46.9 nm eliminates the need for a matrix for ablation and ionization as used in UV LDI MSI.

The EUV laser-created plasmas are also fundamentally different from those created by nanosecond ultraviolet LDI, thereby impacting the characteristics of the ablation. In visible/ultraviolet laser ablation, when the laser fluence is sufficient to ablate the material, the subsequent interaction is dominated by absorption of the laser radiation by inverse bremsstrahlung in the region of the plasma where the electron density approaches the critical density (*n*_ec_∼10^22^ cm^−3^ for *λ*=333 nm). Therefore, once the plasma is created, the ultraviolet laser pulse can no longer reach the sample. However, in spite of this, the sample continues to ablate because heat is conducted from the critical electron density plasma region into the sample. In this heat-driven process ionization of atoms and molecules in the material is dominated by collision with free electrons heated by inverse bremsstrahlung, a process that leads to increased molecular fragmentation. In contrast for EUV light, the critical density is larger than solid density (for *λ*=46.9 nm *n*_ec_=5 × 10^23^ cm^−3^). Therefore, the heating of free electrons by bremsstrahlung absorption is significantly reduced and the plasma is transparent to the incident EUV laser light. Instead, the EUV laser light is predominantly absorbed by photoionization directly on the sample. The absence of significant thermal ablation processes is evident in the AFM profiles of Fig. 1b, which are very smooth and show no damage beyond the ablated region. The mechanisms of ionization are also distinct from those in ultraviolet laser-created plasmas. The EUV photons can ionize any molecule, making photoionization the dominant ionization mechanism over electron impact ionization^21^. At the low fluences necessary to achieve nanoscale spatial resolution, most ions are singly charged, as observed for alanine and Nile red in Fig. 2.

The high 3D localization of the laser/matter interactions of EUV TOF and its superior sensitivity that produces mass spectra from zl volumes open new opportunities to visualize molecular composition at the nanoscale. Optimization of the interaction of the EUV laser light and organic materials in combination with post-ablation ionization^22^ will contribute to extend the mass range. This, coupled with the superior 3D nanoscale resolution, will maximize the potential of EUV TOF MSI for life science applications by enabling, for example, nanoscale compositional imaging of intracellular structure^23^, and the study of chemical interactions between a single microorganism and its host^5^.

## Methods

### Samples and ablation conditions for each experiment

The samples used for the experiments were single or bilayers of organic compounds deposited by thermal evaporation onto a glass substrate coated with a 15–30-nm thick conductive ITO layer. Thin films of polymethyl methacrylate with thickness varying between 60 and 350 nm spun over the ITO-covered glass substrates were used to assess the shape and volume of the ablation crater for each of the laser fluence conditions. The sequence of AFM crater profiles shown in Fig. 1b was obtained by placing the sample at the first-order focus of the ZP and by attenuating the laser beam intensity by photoionization of argon in a variable pressure gas cell. Instead, the craters in Fig. 1a and Supplementary Fig. 2a were obtained by ablating the sample when placed at the third-order focus of the ZP. The use of the third-order focus reduces the size of the ablation spot as a result of the ZP higher numerical aperture, and attenuates the laser fluence by 9 × due to the reduction in the ZP efficiency^19^. The laser fluence necessary to ablate each of the craters was estimated from the FWHM diameter of the AFM profiles of Fig. 1b and from the laser pulse energy measured using a calibrated photodiode.

The sample used for the 2D composition imaging experiments was fabricated by patterning a trench in a ∼120-nm thick resist film (Shipley 1818) using photolithography and standard processing. The removal of the resist in the trench exposed the ITO-coated glass substrate. The ion images of Fig. 4 were obtained using single-shot laser ablation events with an elliptical laser spot with a FWHM minor axis of 300 nm and a FWHM major axis of 500 nm. The fluence at the sample was estimated to be ∼0.43 J cm^−2^. At this fluence, the depth of the ablation crater in the resist was 40 nm.

The sample used for the 3D imaging experiments was prepared by spinning a solution in which the *M. smegmatis* was diluted onto the ITO-covered glass substrate. A microscope image of the region of the sample interrogated is shown in Fig. 5c. Mass spectra were acquired from four consecutive single-shot ablation events on the same sample spot at a laser fluence of ∼0.5 J cm^−2^. Then, the sample was displaced with respect to the laser focal spot by 300 nm and the process was repeated. Each ablation event defined a voxel 0.3 × 0.3 × 0.08 μm^3^. The ion images of Fig. 5 plot iso-lines showing the distribution of two of most salient fragments with *m*/*z* 70.1 and 81.1.

### Data analysis

The analysis of the mass spectra was carried out using a code developed with the scientific software MATLAB that allows for mass calibration, spectra analysis with background subtraction, peak identification and image processing. All mass spectra contained signatures from singly charged parent molecules (*Z*=1).

For imaging, we used a threshold of 3σ the noise level to subtract noise and calculate the intensity under selected peaks in the mass spectrum. The depth profiles of Fig. 3 were constructed as follows. Selected peak areas of alanine corresponding to *m*/*z*≤90 and of Nile red with 90<*m*/*z*≤319 were summed up for each ablation event. A mean analyte signal amplitude was obtained by averaging over a matrix of 4 × 4 ablation events in each layer. Since the ablated volume decreases with every consecutive ablation event following a sub-linear behaviour, the mean peak amplitude for each analyte was normalized to the ablated volume and maximum mean analyte signal. The normalized mean amplitude versus depth is plotted in Fig. 3. The error bar on each point is the variance of the values of the amplitude.

A similar analysis was used to construct the 2D ion images of Fig. 4. In this case, four ion peaks from the resist at *m*/*z* 74.0, 77.1, 91.1 and 121.1 were selected and their amplitudes were weight averaged.

## Author contributions

C.S.M. and D.C.C. conceived the EUV laser ablation mass spectrometry imaging concept. J.F., I.K., J.J.R., C.S.M., F.D., E.R.B and D.C.C. contributed to the design of the instrument; J.F. and I.K. constructed the instrument and wrote the software used for control and data acquisition and analysis; M.W. developed the power supplies; and F.D., J.F. and I.K. prepared samples and collected the data. J.J.R. developed the EUV laser and W.C. and E.H.A. fabricated the diffractive optics. I.K. and J.F. analysed the data and developed the imaging codes. C.S.M. led in the writing of the manuscript and all authors contributed.

## Additional information

**How to cite this article:** Kuznetsov, I. *et al*. Three-dimensional nanoscale molecular imaging by extreme ultraviolet laser ablation mass spectrometry. *Nat. Commun.* 6:6944 doi: 10.1038/ncomms7944 (2015).

## Supplementary Material

Supplementary Information Supplementary Figures 1-2, Supplementary Table 1, Supplementary Discussion and Supplementary References
