# Supplementary material for: Three-dimensional nanoscale molecular imaging by extreme ultraviolet laser ablation mass spectrometry
Source: Nat Commun. 2015 Apr 23;6:6944. doi: 10.1038/ncomms7944 (PMC4423227; doi:10.1038/ncomms7944)
Supplement: Supplementary Information — Supplementary Figures 1-2, Supplementary Table 1, Supplementary Discussion and Supplementary References [file ncomms7944-s1.pdf]

SUPPLEMENTARY MATERIAL

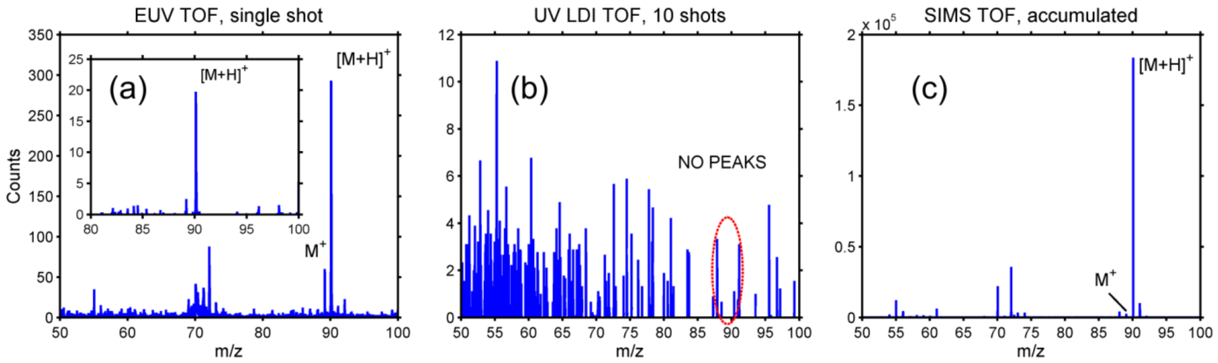

Supplementary Figure 1. Mass spectra of a ~120 nm thick layer of alanine obtained with (a) EUV TOF, (b) UV LDI TOF and (c) SIMS TOF. The M$^+$ and [M+H]$^+$ ion peaks are identified. The spectrum in (a) was obtained from an ablated analyte volume of 12.8 aL, and the one in the inset from a 50 zL volume.

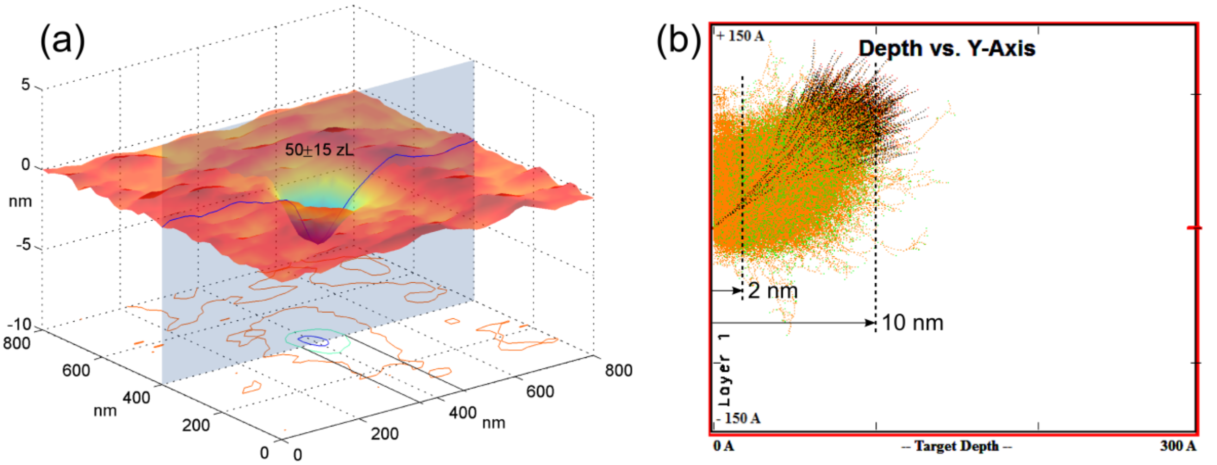

Supplementary Figure 2. a) Atomic force microscope image of a 50 zL crater ablated in polymethyl methacrylate. The blue and orange contour lines show the profiles at 10 and 90% of the crater's depth. At the same irradiation conditions, a crater ablated in alanine produced the mass spectrum shown in the inset of Supplementary Figure 1a. b) TRIM program simulation of a collision cascade as a result of 10 keV Bi$^+$ ion (1000 ions total) interacting with a solid carbon target. The primary ions are incident at 45°. Cascading happens within ~10 nm. The black lines show the primary ion trajectories with red dots at the end, the orange lines represent the displaced atoms (more densely distributed within 2 nm of probe depth) and the green dots show the end of the trajectories for the displaced atoms.

Supplementary Table I. Parameters used in the calculation of sensitivity and level of fragmentation for EUV TOF and SIMS TOF

ALANINE	EUV TOF (First results)	SIMS-TOF
**# of primary particles (dose)**	$3.9\ 10^{6}$	$4.1\ 10^{8}$
**# of secondary ions at 90 m/z**	80	$1.76\ 10^{6}$
**Area probed (µm^2)**	$4.5\ 10^{-2}$	$2.25\ 10^{4}$
**Depth probed (µm)**	$3.5\ 10^{-3}$	$2\ 10^{-3}$
**Volume probed (aL)**	0.05	$4.5\ 10^{4}$
**Sensitivity (amol)**	0.01	0.4
**Ion yield, normalized to probed volume (L^{-1})**	$4\ 10^{14}$	$8.5\ 10^{10}$
**Level of fragmentation**	1.1	1

Supplementary discussion

Comparison of EUV TOF and other mass spectrometry methods

EUV TOF brings new opportunities to MSI by making possible to assess molecular composition in 3D at nanoscale dimensions with high sensitivity. To contrast EUV TOF with leading molecular mass spectrometry methods - ultraviolet laser desorption ionization time of flight mass spectrometry (UV LDI TOF) and static secondary ion mass spectrometry (SIMS TOF) - we conducted experiments in which the same alanine sample was analyzed by the three methods. The SIMS TOF results were obtained by Evans Analytical Group (EAG) using a ION TOF instrument TOF.SIMS 5[1]. For the UV LDI TOF experiments we used a Bruker Ultraflex 2 mass spectrometer without applying a matrix for ionization enhancement to simulate the conditions of EUV TOF.

The alanine mass spectra obtained from each method are shown in Supplementary Figure 1. Comparison between EUV TOF and SIMS TOF reveals the presence of the parent analyte ions, $[M+H]^+$, the radical $M\cdot^+$ and similar fragments' distribution. The main difference is that the aforementioned peaks in the EUV TOF spectrum are well resolved even when the information is extracted from the single shot ablation of a crater with a volume of 50 zL (Supplementary Figure 1 inset). Instead the SIMS TOF spectrum was obtained by scanning 20 times an area of 150×150 µm^2 with a 5 µm diameter Bi_3^+ primary ion beam. The atomic force microscope image

of the 50 zL crater ablated in PMMA as the same conditions of alanine is shown in Supplementary Figure 2a.

The sensitivity of a mass spectrometry method is calculated as the ratio of the ablated/sputtered analyte mass in moles to the counts within the area of the dominant parent ion, i.e. $[M+H]^+$. Using the alanine spectrum in the inset that shows $[M+H]^+$ peak is well above the noise level we calculate the sensitivity of EUV TOF to be 0.01 amol.

A similar calculation was performed for SIMS TOF. Since the depth of the analyte region probed by SIMS TOF was not provided by EAG, we made use of commercial software (TRIM – Transport of Ions in Matter, based on the Monte-Carlo simulation method, http://www.srim.org/) to estimate the ion/analyte interaction region and the depth from which secondary ions are generated. Because the primary ions used for sputtering were relatively small clusters of Bismuth – Bi_3^+ at energies of 30 keV - we modeled this interaction as that of a single Bi projectile of 10 keV energy. Z. Postawa et al showed that multi-atomic inorganic projectiles dissociate upon impact, hence the energy is divided equally among its species[2]. We also simplified the target to be pure carbon. A plot of the primary ion trajectories and the displaced atoms as a result from the collision cascade process in SIMS TOF is shown in Supplementary Figure 2b. It has been shown secondary ions originate from the top few monolayers of the analyte's surface[3], i.e. ~2 nm. This assumption correlates well with our modelling. The displaced target atoms, indicated by the orange traces in Supplementary Figure 2b, are most dense within the top 2 nm of the carbon sample. However, the collision cascade contributing to surface ejection of sputtered species, occupies deeper layers, ~10 nm, as also shown in Supplementary Figure 2b[4]. We used a conservative probe depth of ~2 nm for SIMS TOF to calculate the alanine mass from which ions originate in the spectrum of Supplementary Figure 1c. This value is a lower estimate for the penetration depth because alanine has lower density than carbon. A depth of 2 nm corresponds to ~4 monolayers of alanine. The sensitivity of SIMS TOF is calculated to be 0.4 amol, which is 40× lower than that of EUV TOF. Supplementary Table I summarizes the results of the sensitivity analysis.

The model simulation also shows that for the conditions of the SIMS TOF experiment, the depth resolution in MSI cannot be better than ~10 nm, the extend of the collision cascade process where the analyte is damaged. The work by D. Rading et al[4] showed that the type of clusters and their energy can be selected to reduce the depth of the 'damaged' region in SIMS TOF. Notice that in contrast to SIMS TOF, there is no damage to the sample outside the ablated crater in EUV TOF.

The differences between EUV TOF and UV LDI TOF can be accounted for by analyte absorption at the different wavelengths. Alanine has minimum absorption at λ=333 nm[5], thus the mass spectrum mainly contains spurious peaks even after 10 laser shots were accumulated. Instead in EUV TOF organic solids are strong absorbers at λ=46.9 nm, making it possible to controllably ablate and efficiently ionize the analyte.

The level of fragmentation was also calculated from the EUV TOF and SIMS TOF spectra of Supplementary Figure 1 as the ratio of total counts within $[M+H]^+$ to the total number of counts in the spectrum within the m/z range 50-90, namely the peaks at m/z 55. 70, 72 and 89. For EUV TOF we used the mass spectrum obtained from the ablated 12.8 aL crater. The level of

fragmentation of EUV TOF is found similar to SIMS TOF. Supplementary table I summarizes the results of this comparison.

In summary, based on the results of the analysis of the same alanine sample it is shown that EUV TOF has higher sensitivity and similar level of fragmentation. EUV TOF also has significantly smaller 3D localization. In comparison with UV LDI TOF, the results show EUV light is naturally suited for mass spectrometry of organic analytes due to the high absorption of these materials at λ=46.9 nm.

Supplementary References

1 http://www.eag.com/. *Evans Analytical Group*, 2014).

2 Postawa, Z. *et al.* Microscopic insights into the sputtering of Ag{111} induced by C-60 and Ga bombardment. *J. Phys. Chem. B* **108**, 7831-7838, doi:10.1021/jp049936a (2004).

3 Russo, M. F. & Garrison, B. J. Mesoscale energy deposition footprint model for kiloelectronvolt cluster bombardment of solids. *Anal. Chem.* **78**, 7206-7210, doi:10.1021/ac061180j (2006).

4 Rading, D., Moellers, R., Cramer, H. G. & Niehuis, E. Dual beam depth profiling of polymer materials: comparison of C-60 and Ar cluster ion beams for sputtering. *Surf. Interface Anal.* **45**, 171-174, doi:10.1002/sia.5122 (2013).

5 Caroline, M. L., Sankar, R., Indirani, R. M. & Vasudevan, S. Growth, optical, thermal and dielectric studies of an amino acid organic nonlinear optical material: L-Alanine. *Materials Chemistry and Physics* **114**, 490-494, doi:10.1016/j.matchemphys.2008.09.070 (2009).
